# Primary Ciliary Dyskinesia Complicated by Stroke in an Elderly Male: A Case Report

**DOI:** 10.1002/rcr2.70163

**Published:** 2025-03-20

**Authors:** Ali Gohar, Bilal Ahmed, Asim Ali, Maryam Ilyas, Momina Masroor, Ayesha Ayman, Masab Ali, Muhammad Husnain Ahmad

**Affiliations:** ^1^ Department of Medicine Lahore General Hospital Lahore Punjab Pakistan; ^2^ Department of Medicine Pakistan Kidney and Liver Institute and Research Centre Lahore Punjab Pakistan; ^3^ Department of Internal Medicine Punjab Medical College Faisalabad Punjab Pakistan; ^4^ Department of Medicine Satkynbay Tentishev Asian Medical Institute Kant Kyrgyzstan

**Keywords:** cerebrovascular accident, dextrocardia, ischemic stroke, Kartagener syndrome, primary ciliary dyskinesia, situs inversus

## Abstract

Kartagener syndrome (KS) is an uncommon hereditary disorder, featuring situs inversus, chronic sinusitis, and bronchiectasis. Our case report presents a 60‐year‐old Asian male with KS who was incidentally diagnosed with KS upon presenting with an ischemic cerebrovascular accident (CVA). The patient had a longstanding history of poorly controlled type 2 diabetes mellitus and hypertension, presenting with acute right‐sided weakness and speech impairment. His history of recurrent respiratory infections and infertility, combined with family findings of dextrocardia and male infertility, suggested KS. On physical examination, the patient exhibited signs of dextrocardia. Imaging confirmed situs inversus and dextrocardia, while neurological evaluation revealed an embolic stroke in the left middle cerebral artery territory. Certain cardiovascular anomalies in KS may affect stroke risk; however, their co‐occurrence in the patient appears to be coincidental, given the patient's baseline risk for stroke. A high‐resolution chest CT demonstrated bronchiectasis and carotid Doppler ultrasound identified atherosclerotic plaques, likely contributing to the stroke. This case highlights the need for heightened awareness of neurological events, such as stroke, in patients with KS. Cardiovascular risk factors, compounded by the complexity of the syndrome, require prompt evaluation and multidisciplinary care to prevent severe complications.

## Graphical abstract

This report presents a unique case of primary ciliary dyskinesia, complicated by an ischemic cerebrovascular accident (CVA) in a patient with Kartagener syndrome. It underscores the diagnostic complexities and emphasises the need for prompt, multidisciplinary intervention to mitigate the risk of severe neurological outcomes.

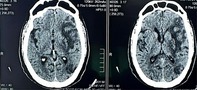

## 1 Introduction

Kartagener syndrome (KS) is a rare genetic condition, inherited in an autosomal recessive manner, and falls under the broader category of primary ciliary dyskinesia (PCD) [1, 2]. PCD arises from structural and functional abnormalities in motile cilia, which impair mucociliary clearance, leading to recurrent respiratory infections, chronic sinusitis, and bronchiectasis [3]. In addition to these manifestations, defective ciliary function during embryogenesis can disrupt left–right axis determination, resulting in situs inversus and a spectrum of congenital anomalies, including cardiovascular malformations. These developmental irregularities highlight the systemic implications of PCD beyond its impact on the respiratory system [3]. Hence, KS is typified by a triad of clinical features: situs inversus, chronic sinusitis, and bronchiectasis [1, 2]. Globally, KS affects approximately 1 in 10,000–40,000 people, with symptoms often emerging in early childhood as recurrent respiratory infections and sinusitis due to poor mucus clearance [1, 4].

While respiratory symptoms, including bronchiectasis, are the most prominent manifestations of KS, the co‐occurrence of situs inversus complicates the diagnosis and clinical management [2]. The reversal of internal organs adds to the complexity, particularly when patients present with additional complications such as cardiovascular abnormalities or neurological events like stroke [3]. Aside from situs inversus, other kinds of congenital cardiac and vascular anomalies, such as atrial or ventricular septal defects and tetralogy of Fallot, may be seen in patients with KS [3]. Some of these anomalies are known to predispose to thromboembolic events, as discussed by Giang et al. [5]. These anomalies can increase the risk of embolic strokes through mechanisms such as paradoxical embolism or turbulent flow, especially in the presence of right‐to‐left shunts.

This report presents a unique case of PCD, complicated by an ischemic cerebrovascular accident (CVA) in a patient with KS. It underscores the diagnostic complexities and emphasises the need for prompt, multidisciplinary intervention to mitigate the risk of severe neurological outcomes.

## 2 Case Report

A 60‐year‐old Asian male with a known history of poorly controlled type 2 diabetes mellitus for 15 years and hypertension for the past year presented to the emergency department of a tertiary care hospital with sudden onset of right‐sided weakness and aphasia that had persisted for 1 day. The neurological symptoms were progressive, without any associated fever, seizures, trauma, or substance abuse. His medical history revealed poor compliance with oral antihypertensive and antidiabetic medications. Despite being married for 20 years, the patient and his wife had been unable to conceive, raising concerns about underlying infertility. Further inquiry revealed a history of recurrent respiratory tract infections, a key feature that prompted the consideration of a rare underlying syndrome.

His family history was significant, with three of his six siblings, all male, experiencing infertility, and three brothers, including him, having dextrocardia. This familial clustering, alongside his clinical presentation, suggested an autosomal recessive inheritance pattern indicative of KS, a rare subset of PCD.

Initial physical examination revealed an obese patient with a Glasgow Coma Scale (GCS) score of E4V1M6, reactive pupils, and an upward plantar reflex on the right side. Cardiovascular examination was notable for dextrocardia, as evidenced by the apex beat being palpated on the right side of the chest. Abdominal ultrasound revealed situs inversus, with the liver located on the left side and other abdominal organs in a mirrored orientation. Electrocardiographic findings showed a sinus rhythm with no evidence of atrial fibrillation, ruling out a cardioembolic source for the stroke. A 2D echocardiogram confirmed dextrocardia but did not reveal other congenital cardiac or vascular anomalies, such as atrial or ventricular septal defects or tetralogy of Fallot, which are known to predispose to thromboembolic events.

**FIGURE 1:**
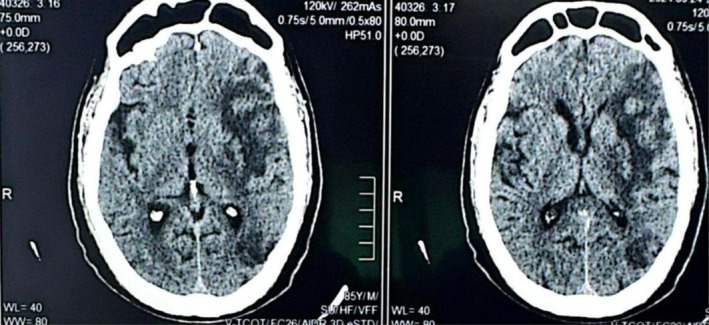
CT brain showing hypodense area in left temporal lobe, indicating an embolic infarct in the left middle cerebral artery (MCA).

**FIGURE 2:**
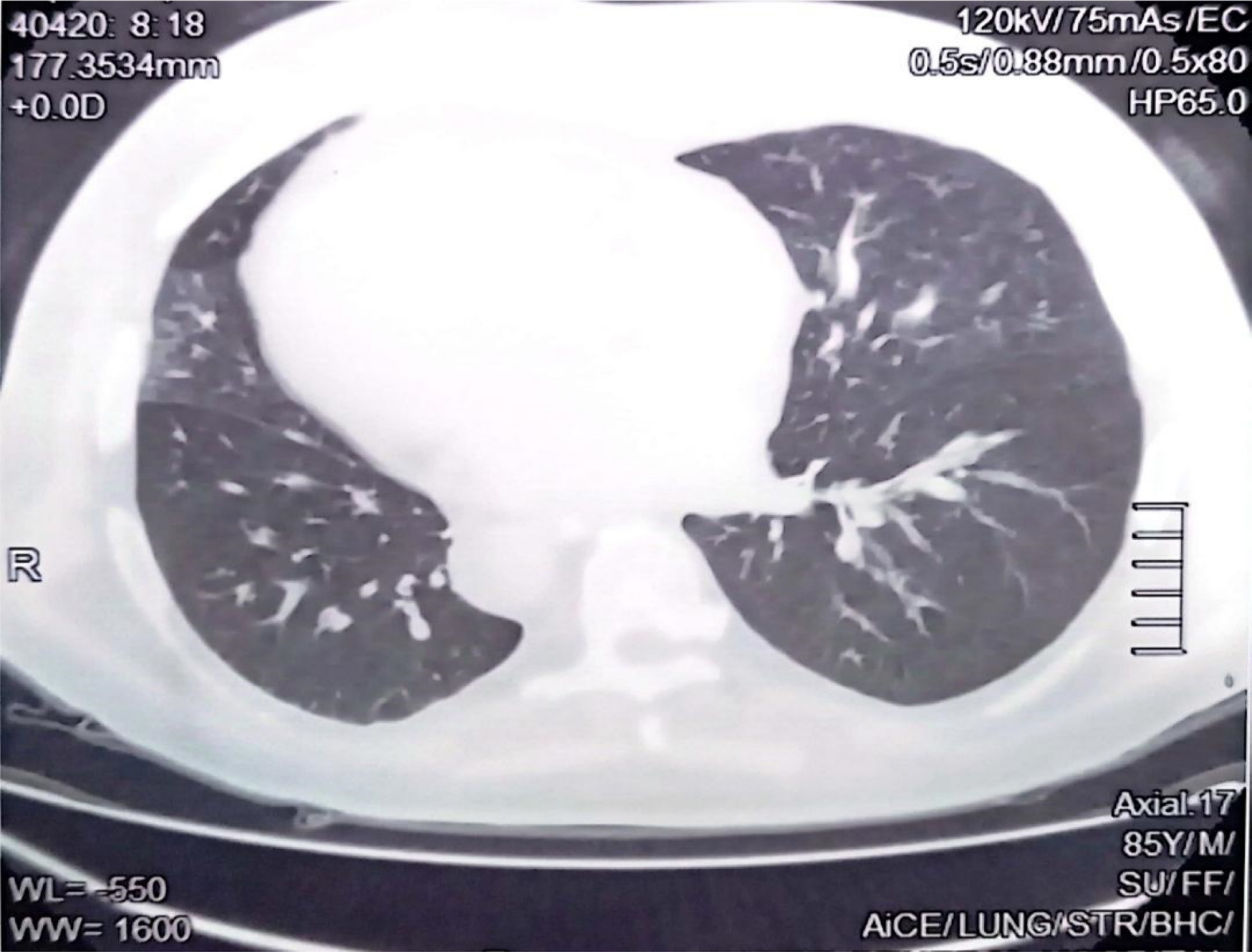
A high‐resolution CT (HRCT) scan of the chest demonstrated focal central bronchiectasis, ground‐glass opacities and pneumatocele.

Neurological imaging with a CT brain scan identified a hypodense area in the left temporal lobe, indicating an embolic infarct in the left middle cerebral artery (MCA) territory, which explains the patient's right‐sided weakness and aphasia (Figure 1). A high‐resolution CT (HRCT) scan of the chest demonstrated focal central bronchiectasis, ground‐glass opacities and pneumatocele, further supporting the diagnosis of KS (Figure 2). Carotid Doppler ultrasound revealed bilateral atherosclerotic plaques, indicating significant cardiovascular risk factors that likely contributed to the ischemic cerebrovascular accident (CVA) (Table 1). Laboratory investigations showed mildly elevated triglycerides at 150 mg/dL and serum cholesterol levels of 136 mg/dL (Table 2). The patient was managed with antiplatelet therapy, statins, and strict control of his hypertension and diabetes. He was discharged with a multidisciplinary follow‐up plan, including neurology, pulmonology, and cardiology consultations to address his cerebrovascular and systemic comorbidities.

**TABLE 1:** Carotid Doppler ultrasound and echocardiography findings.

	Right carotid	Left carotid
CCA intima	7 mm	7 mm
CCA velocity	56 cm/s	53 cm/s
ICA velocity	44 cm/s	39 cm/s

Echocardiography	mm	Range
Aortic root	26	20–37
Left atrium	46 × 76	20–39
LV end diastole	48	42–56 (male)
LV end systole	35	30–40 (male)
IVS thickness	16	6–10 (male)
LWPW thickness	16	6–10 (male)
LVEF	60%	> 55%
FS	30%	30%–45%
Mitral regurgitation	Positive	
Tricuspid regurgitation	Positive	
PASP	25 mm Hg	

**TABLE 2:** Basic metabolic panel.

Test (s)	Result (s)	Reference range (s)
Haemoglobin	15.1 g/dL	13.0–16.5 g/dL
Total leukocyte count (TLC)	16.4 × 10^3^/μL	4.0–11.0 × 10^3^/μL
Platelets	169 × 10^3^/μL	150–400 × 10^3^/μL
Creatinine	1.19 mg/dL	0.4–1.3 mg/dL
Urea	25 mg/dL	15–40 mg/dL
Bilirubin	0.8 mg/dL	0.2–1.0 mg/dL
AST	28 U/L	Upto 40 U/L
ALT	22 U/L	Upto 40 U/L
ALP	145 U/L	42–306 U/L
Triglycerides	150 mg/dL	< 150 mg/dL
Cholesterol	136 mg/dL	< 200 mg/dL
Sodium	141 mmol/L	135–155 mmol/L
Potassium	4.3 mmol/L	3.5–5.1 mmol/L
Chloride	101 mmol/L	98–107 mmol/L
Prothrombin time (PT)	11 s	11–14 s
APTT	30 s	26–32 s
INR	1.18	< 1 s

## 3 Discussion

Diagnosing KS can be challenging due to the variability in clinical presentation, overlap with other conditions, and limitations in access to specialised diagnostic tools in many settings. Although consensus guidelines for the diagnosis and management of PCD have been established, including recommendations by Shapiro et al. [6] and Barbato et al. [7], these guidelines may not be universally applied, particularly in resource‐limited settings. Diagnostic methods now include nasal nitric oxide measurements, high‐speed video microscopy, genetic testing, and electron microscopy to evaluate ciliary structure and function. However, the complexity of these techniques and their limited availability contribute to delays and challenges in diagnosing PCD and its subset, KS. Hence, in most cases, confirmation occurs years after symptom onset, sometimes in adulthood [2]. Our patient's diagnosis of KS was incidental, following an ischemic cerebrovascular accident (CVA).

To the best of our knowledge, this is the first reported association of KS with ischemic stroke. The co‐occurrence of KS and ischemic stroke in this patient underscores the need to consider both syndromic features and individual baseline risk factors. As an elderly male with poorly controlled hypertension, type 2 diabetes mellitus, obesity, and carotid artery disease, he was undoubtedly at high risk for cerebrovascular events, independent of KS. While KS‐related cardiovascular anomalies might contribute to stroke risk, the available evidence does not support a direct link in this case. This distinction emphasises the importance of addressing modifiable risk factors and maintaining comprehensive care for patients with rare syndromes.

KS, being a congenital and inherited disorder, is typically diagnosed in early childhood or adolescence [4], when recurrent respiratory infections and chronic sinusitis become apparent. However, in some cases, particularly in resource‐limited settings or when clinical features are mild or atypical, diagnosis may be delayed well into adulthood. In our patient, the diagnosis was made at 60 years of age, likely due to the lack of early recognition of the characteristic symptoms of KS, such as recurrent respiratory tract infections and situs inversus, and the absence of advanced diagnostic tools during his early years. This discrepancy in age highlights the importance of increased clinical suspicion, especially in patients who arrive with infertility, recurrent respiratory infections and dextrocardia.

In contrast to a study by Vaid et al. [1], where no vascular complications were noted, our patient had bilateral atherosclerotic plaques on carotid Doppler ultrasound, complicating the clinical picture, suggesting the need for additional research.

Though consanguinity is often associated with KS, as noted by Nisrine et al. [2], our patient had no consanguineous family history, but three of his six male siblings experienced infertility, and several shared the characteristic of dextrocardia. This unique family history underscores the importance of genetic testing in such cases as well.

While studies by Hyseni et al. [8] and Zhang et al. [9] have reported cases of neurological manifestations in patients with situs inversus and KS, these instances primarily describe co‐occurrences rather than confirmed associations. For example, Zhang et al. [9] documented a case of KS and Moyamoya syndrome but emphasised the need for further studies to confirm any association and elucidate potential mechanisms. Similarly, Hyseni et al. [8] reported acute spinal cord ischaemia in a patient with situs inversus totalis; however, the aetiology of situs inversus was not established as KS, and no evidence was provided to link the neurological manifestation with the underlying anatomical anomaly. These cases underscore the complexity of interpreting such co‐occurrences and highlight the need for cautious interpretation, as mechanisms of disease may vary significantly.

This case presents the rare occurrence of KS complicated by an ischemic cerebrovascular accident, emphasising the importance of increased clinical vigilance in patients with recurrent respiratory infections, infertility and dextrocardia. The combination of systemic and neurological symptoms in this patient underscores the necessity for thorough evaluation and a multidisciplinary approach in managing rare genetic syndromes with intricate clinical presentations.

## Author Contributions

**Ali Gohar:** conceptualisation, data curation, project administration, supervision, validation, visualisation, writing – original draft, writing – review and editing. **Asim Ali:** project administration, supervision, validation, visualisation, writing – original draft, writing – review and editing. **Bilal Ahmed:** project administration, supervision, validation, visualisation, writing – original draft, writing – review and editing. **Ayesha Ayman:** validation, visualisation, writing – original draft, writing – review and editing. **Maryam Ilyas:** validation, visualisation, writing – original draft, writing – review and editing. **Momina Masroor:** validation, visualisation, writing – original draft, writing – review and editing. **Masab Ali:** validation, visualisation, writing – original draft, writing – review and editing. **Muhammad Husnain Ahmad:** validation, visualisation, writing – review and editing.

## Ethics Statement

The authors declare that appropriate written informed consent was obtained for the publication of this manuscript and accompanying images.

## Conflicts of Interest

The authors declare no conflicts of interest.

## Data Availability

Data and materials are available upon reasonable request from the corresponding author.
